# Sleep and Military Members: Emerging Issues and Nonpharmacological Intervention

**DOI:** 10.1155/2013/160374

**Published:** 2013-07-14

**Authors:** Cary A. Brown, Robyn Berry, Ashley Schmidt

**Affiliations:** Department of Occupational Therapy, Faculty of Rehabilitation Medicine, University of Alberta, 2-64 Corbett Hall, Edmonton, AB, Canada T6G 2G4

## Abstract

*Background*. Many individuals who work in the military experience sleep deficiency which presents a significant problem given the nature of their work. The cause of their sleep problems is likely multifactorial, stemming from the interplay between their personal health, habits and lifestyle juxtaposed with the stress of their military work such as emotional and physical trauma experienced in service. *Objective*. To present an overview of sleep deficiency in military members (MMs) and review of nonpharmacological treatment options. *Discussion*. Although there are a number of promising nonpharmacological treatment options available for people working in the military who experience problems sleeping, testing interventions within the context of the military are still in the early stages. Further research utilizing rigorous design and standardized, context appropriate outcome measures is needed to help treat this burgeoning problem.

## 1. Introduction 

The objective of this report is to present an overview of the significance of sleep deficiency (SD) in military members (MMs) and highlight the current state of the evidence for non-pharmacological, evidence-based sleep interventions (NPSIs) [1]. 

## 2. Background

Sleep deficiency is a growing problem for people of all ages and significantly impacts many aspects of an individual's life. It is now emerging that persons in the military experience even greater challenges with sleep deficiency than most members of the general public [2–4]. Sleep deficiency is defined by the National Centre on Sleep Disorders Research [5] as, “…too little sleep, poor quality sleep or sleep problems including diagnosed sleep disorders.” Restorative sleep is needed for optimal brain function and overall health. Individuals with sleep deficiency have an increased risk of age-related illnesses such as diabetes, hypertension, obesity, and memory loss [6]. Emerging studies have also identified that SD is not simply a symptom of an existing illness but also acts as a risk factor, increasing the likelihood of developing health conditions such as diabetes, hypertension [7], impaired judgment and depression [8], and chronic pain [9].

### 2.1. Prevalence of Sleep Deficiency for Military Members

Sleep deficiency is common during and after deployment for MM [10, 11]. Research reports that over 75% of deployed military members rated their sleep quality as significantly worse than their sleep prior to deployment [4]. The literature suggests that the most common sleep disorders found in a MM are sleep breathing disorders and movement disorders [11, 12]. As well, insomnia is a common, unrelenting, and debilitating complaint for those returning from military deployment [10]. 

Military members have higher rates of life and job dissatisfaction, depression, and negative mental health when compared to the overall working population [2]. Sleep deficiency is a common feature of many of conditions that veterans live with as documented in a 2011 Canadian survey [3]. This survey found that 90% of veterans were diagnosed with at least one physical health condition, 50% reported at least one mental health condition, and almost 70% had between four to six physical and mental health conditions. Similar issues have been identified in American MM [4]. 

### 2.2. Etiology of Sleep Deficiency in MM

Sleep deficiency in MM is likely caused by the interaction of a number of factors. These factors include environmental and social influences, comorbid physical and mental health, individual habits, beliefs and lifestyle choices, preenlistment sleep quality as well as emotional and physical trauma experienced in service. For example, physical health could be changed by practices such as physical overtraining, dietary routines, and prolonged physiological arousal consequent to hyper-vigilance. Chronic pain, ruminations such as thinking about the traumatic event, general worries, and substance misuse [33–36] also contribute to the development of SD.

There are three main routes that MM can acquire sleep deficiency: presence of a preexisting sleep conditions such as sleep apnea, during their military service, or due to a traumatic event or injury during combat. New recruits may also bring poor sleep hygiene practices with them when they join the military. These practices may include alcohol use, smoking, irregular hours, substance misuse to self-medicate for sleep/wake cycle disruption, and extended electronic device use. Some military members are predisposed to SD due to physical inactivity, lifestyle choices, substance misuse, exposure to extreme environments (noise, heat, and light), beliefs about sleep and activity, comorbid conditions that contribute to SD (e.g., pain), and certain medications [33]. Weight, smoking, and alcohol use are all risk factors for sleep apnea. Sleep apnea is highly prevalent in MM with prevalence rates of 37.5% in active MM reported in 2005 [37, 38] and as high as 62.7% in a 2013 study of 110 MMs recently returned from combat [39].

Posttraumatic stress disorder (PTSD) is a growing concern for MM and a relationship between PTSD and SD problems (such as fragmented sleep, insomnia, nightmares, night terrors, and night time anxiety attacks) has been well documented [34–36]. Preexisting SD is believed to be a risk factor for a MM to develop PTSD and restorative sleep appears to serve a protective function [7]. Post-traumatic stress disorder appears to have a bidirectional relationship with SD. Chronic sleep deficiency may be a risk factor for developing PTSD and symptoms of PTSD lead to the development of SD [35, 40, 41]. This is congruent with the apparent bidirectional relationship demonstrated between a range of medical and psychiatric disorders and insomnia, as insomnia may reciprocally predispose patients to relapse or exacerbate a recurrence of the condition [41]. 

A final area to highlight is the relationship between mild traumatic brain injury (mTBI) and sleep [42, 43]. The number of reported mTBI incidents and concussions in youth is growing [44] and active, apparently fit, young people bring these pre-existing injuries with them into military service. There is also an increased incidence of head trauma, mTBI, and blast related concussion injuries in active MM [45]. Persons with TBI demonstrate significantly lower levels of evening melatonin production, reduced slow wave sleep, and elevated depression all of which disrupt circadian regulation and restorative sleep [46]. A study of 26 adults with mTBI found that light, nonrapid eye movement (NREM) sleep was significantly higher and REM stage sleep was lower than controls [42]. Because REM sleep is important for cognitive function, memory, and new learning, these findings are particularly of concern given the high cognitive demand of many military roles. This is an important new area to explore.

Once enlisted, existing SD is compounded and new sleep problems emerge as a consequence of the environmental, physical, and psychological challenges military service presents to restorative sleep [33]. These challenges include communal sleeping, training, and field action that is highly alerting and does not allow for a “wind-down” period prior to sleep and psychologically unsettling events. A study of British soldiers found that 19.5% snored and this behavior can have a serious negative impact on the sleep of others in communal settings [38]. Additionally, the appropriate timing of daylight exposure needed for circadian regulation may not be possible. The military can also promote a culture in which self-care activities like sleep are believed to have little standing value and demonstrate a personal shortcoming such that the individual is perceived to lack the stamina required for the demands of active service. As Bray [10] points out in this culture the acceptance of sleep deprivation and efforts to function in a sleep deprived state can be seen as indelibly heroic.

### 2.3. How Sleep Deficiency Impacts Military Members

There is strong evidence that SD significantly impacts cognitive, emotional, and physical functioning in MM [10]. Attention, alertness, memory, routine task performance, and physical performance are all negatively affected by SD [47]. For example, MMs who have an average of four to six hours of sleep per night or have not slept in over 16 hours show a noticeable decrease in performance [48]. After four days of sleep deprivation the MM participants in one study were no longer effective and demonstrated impaired decision making and reaction time [47]. Given that these individuals can face life threatening tactical decisions on a daily basis, impaired judgment or performance can have dangerous outcomes [48]. Military members with SD perform similarly on neurocognitive exams to those who had consumed three alcoholic beverages [10]. They also have poorer work effectiveness and subsequently increased accident rates [10]. Research reports that are between 12 and 25% of the most severe military accidents have been linked to fatigue [49]. 

## 3. Interventions for Sleep Deficiency

Current treatments for SD include both pharmacological and nonpharmacological approaches [8, 35, 50]. 

### 3.1. Pharmacological Treatments

Medications for SD in military members are the same as those used with civilians and can include antidepressants, sedatives/hypnotics, and adrenergic antagonists/anticonvulsants. To date, there is sparse research into the outcomes of these medications specific to MMs. This lack can be problematic given the quite unique range of biological, physiological, and emotional stressors MMs are exposed to as compared to the civilian population and more targeted research is indicated. As with the civilian population these interventions need to be used with caution because of side effects and potential to suppress the Rapid Eye Movement (REM) stage of sleep [49]. The benefit of drug therapies in the treatment of SD is controversial and there is limited evidence to support positive outcomes in long-term use [51] and particularly during deployment [52]. In addition, many side effects have been noted with the use of medications with resultant impaired cognition, insight, new learning, and other cortical functions [35]. Long-term use of medication for SD can reduce stages three and four of the sleep cycle which are necessary for physiological restoration and metabolism [51, 53, 54]. Recent guidelines [50] recommend short courses of corrective sleep medication and more long-term strategies of non-pharmacological sleep interventions (NPSIs) for SD. 

The use of Modafinil (a psychostimulant available in the US since 2003) also warrants discussion in this overview. In some areas of the military this drug is used to suppress the normal circadian drive for sleep and foster prolonged states of cognitive arousal. Dongsoo's recent review of the Modafinil literature highlighted that the pharmacological mechanism of the drug is not yet clear and that some studies have identified concerns with potential abuse for recreational purposes, addiction, and the negative consequences of extended periods of sleeplessness on the immune system [55].

### 3.2. Nonpharmacological Interventions (NPSIs)

Little is known about NPSI interventions for SD in the unique context of MM and veterans [9]. A number of psychological and behavioural treatments have been successful to treat insomnia in a range of populations [51]. These include stimulus control therapy, sleep restriction therapy, relaxation training, cognitive therapy, sleep hygiene education, and cognitive behavioural therapy [41, 51]. In the small body of research that exists which focuses on MM and veterans behavioral treatment for insomnia [4, 10], basic sleep hygiene principles [33], bright light therapy for MM experiencing combat-related PTSD [56], and biofeedback [57] have shown to be promising interventions. 

A recent structured critical review of the methodological quality of NPSI research specific to MM [1] employed the Effective Public Health Practice Project (EPHPP) quality assessment tool [58, 59] and the Jadad quality of randomization scale [60]. The review yielded only 21 studies specific to military populations (see Table 1). Imagery Rehearsal Therapy (IRT) alone [13–16], in combination with sleep hygiene education [17–19], and combined with cognitive behavioral approaches (CBAs) [20–23], was the most frequently studied NPSI and demonstrated the strongest overall methodological quality. Other NPSIs, such as multicomponent interventions [24, 25], Biofeedback [12, 26], relaxation [29], Mind Body Bridging [30], Flooding [31], and fitness programs [32] demonstrated only weak to moderate methodological quality and were studied less frequently. Two separate studies of cognitive behavioral approaches [27, 28] were also retrieved and rated as methodologically strong. An important positive finding was that 20 of the 21 studies were rated as strong in the domain of outcome measures. Encouragingly many of the outcome measures used in these studies are relevant and pragmatic for use in clinical settings. Overall research for NPSIs is in the preliminary stage and there is currently limited evidence of efficacy such that policy makers can strategically target service delivery. Further research is required for the effectiveness of NPSIs for military member's sleep disorders before the research can be deemed as being considered conclusive.

## 4. Discussion

There is a clear need for researchers to develop methodologically sound studies to address the unique context and complexity of factors that interact to precipitate and perpetuate SD in MM. An important consideration in any intervention is acceptability to the patient. Reassuringly, there is evidence to suggest MMs are not adverse to NPSIs. For example, Sloberg's [61] survey of 640 active-duty naval personnel found that 61.6% used complementary and alternative medicine (CAM) therapies within the last year. Sloberg also noted that this was comparable to CAM use in the general population. Part of this acceptability is of course the ability to apply an intervention in a simple to apply and nonstigmatizing fashion. However, many of the NPSIs we reviewed were not readily pragmatic, requiring additional training or equipment to implement either during active duty or in the home setting. This is a concern because pragmatic NPSIs that are incorporated into active duty can serve a health promotion and illness preventative function [4]. Additionally, the lack of NPSI outcome studies with a qualitative design is a concern. This is an important gap in the research because qualitative methods add the external validity and deeper insight needed to enhance our understanding of NPSIs as they apply to the complex context of sleep in MM. Finally, there is also a need for research involving both genders of young MM engaged in active duty. Much of the research involves predominantly older male veterans and is nonreflective of the current MM demographic of a younger, more gender-balanced population. For example in Canada, 46.7% of the military population are female and 52.6% of MM are below the age of 39 [2]. 

## 5. Conclusion

The evidence base for interventions for NPSI in MM is still under development and cannot be considered conclusive but can be viewed as promising. NPSIs are widely used, can be highly pragmatic, and are often congruent with principles of health self-management and personal control. Encouragingly, the literature has consistently demonstrated positive NPSI outcomes with no reported adverse effects. This literature highlights emerging and extensive opportunities for both research and clinical applications. It is important to note that while gaps in the extant research and methodological weakness in a study's design may result in a lack of evidence for the specific intervention, it does not follow that the intervention has no merit and should be discarded. Rather there is a clear need for more rigorous research within the unique context of military service. The unmet need to better assess and provide NPSI for SD in military members is of particular concern. The evidence is clear that SD impacts the physical, cognitive, and emotional functioning essential for military members to perform their jobs properly. The mission now is to bridge this research to practice gap so that clinicians and their MM patients can move forward with meaningful and effective sleep interventions.

## Figures and Tables

**Table 1a:** (a) Imagery rehearsal therapy alone or in combination

Imagery rehearsal therapy (i) Strong [13] (ii) Moderate [14–16]	Imagery rehearsal therapy + education (i) Strong [17] (ii) Moderate [18] (iii) Weak [19]	Imagery rehearsal therapy + cognitive behavioral approaches (i) Moderate [20–22] (ii) Weak [23]

**Table 1b:** (b) Other

Multicomponent (i) Moderate [24] (ii) Weak [25]	Biofeedback (i) Moderate [26] (ii) Weak [12]	Cognitive behavioral approaches (i) Strong [27, 28]	Relaxation (i) Weak [29]

Mind body bridging (i) Moderate [30]	Flooding (i) Moderate [31]	Fitness (i) Weak [32]	
